# Advances of traditional Chinese medicine preclinical mechanisms and clinical studies on diabetic peripheral neuropathy

**DOI:** 10.1080/13880209.2024.2369301

**Published:** 2024-06-30

**Authors:** Yuna Zhang, Xianglong Wu, Wenhui Yao, Yadong Ni, Xuansheng Ding

**Affiliations:** a School of Basic Medicine and Clinical Pharmacy, China Pharmaceutical University, Nanjing, China; b Precision Medicine Laboratory, School of Basic Medicine and Clinical Pharmacy, China Pharmaceutical University, Nanjing, China

**Keywords:** Diabetes mellitus, peripheral nervous system diseases, single herbs, self-composed Chinese herbal compound prescription, Chinese patent medicine, inflammatory reaction

## Abstract

**Context:**

Diabetic peripheral neuropathy (DPN) results in an enormous burden and reduces the quality of life for patients. Considering there is no specific drug for the management of DPN, traditional Chinese medicine (TCM) has increasingly drawn attention of clinicians and researchers around the world due to its characteristics of multiple targets, active components, and exemplary safety.

**Objective:**

To summarize the current status of TCM in the treatment of DPN and provide directions for novel drug development, the clinical effects and potential mechanisms of TCM used in treating DPN were comprehensively reviewed.

**Methods:**

Existing evidence on TCM interventions for DPN was screened from databases such as PubMed, the Cochrane Neuromuscular Disease Group Specialized Register (CENTRAL), and the Chinese National Knowledge Infrastructure Database (CNKI). The focus was on summarizing and analyzing representative preclinical and clinical TCM studies published before 2023.

**Results:**

This review identified the ameliorative effects of about 22 single herbal extracts, more than 30 herbal compound prescriptions, and four Chinese patent medicines on DPN in preclinical and clinical research. The latest advances in the mechanism highlight that TCM exerts its beneficial effects on DPN by inhibiting inflammation, oxidative stress and apoptosis, endoplasmic reticulum stress and improving mitochondrial function.

**Conclusions:**

TCM has shown the power latent capacity in treating DPN. It is proposed that more large-scale and multi-center randomized controlled clinical trials and fundamental experiments should be conducted to further verify these findings.

## Introduction

Diabetes mellitus (DM) is a collection of metabolic disorders characterized by incomplete glucose utilization and excessive production due to abnormal gluconeogenesis and glycogenolysis, leading to hyperglycemia (Sacks et al. 2023). In 2021, approximately 537 million adults (20 ∼ 79 years) lived with diabetes. It’s estimated to increase to 643 million by 2030 and 783 million by 2045 (Magliano et al. 2021). Individuals with diabetes are at increased risk of developing complications that commonly affect the heart, blood vessels, eyes, kidneys, nerves, teeth, and gums (Magliano et al. 2021). These complications, especially microvascular (predominantly neuropathy) and macrovascular issues, contribute to diabetes being the fourth leading cause of death in developed countries (Roglic et al. 2005). About 6.7 million deaths worldwide in 2021 were attributed to diabetes-related causes (Magliano et al. 2021). Consequently, effective strategies to combat diabetes complications are urgently needed. Diabetic peripheral neuropathy (DPN), a prevalent and debilitating microvascular complication of type 2 DM (T2DM), affects nearly 50% of patients after 10 years of the disease (Ang et al. 2014). It manifests as a heterogeneous group of symptoms affecting the peripheral nervous system (Iqbal et al. 2018) with various clinical features (Faselis et al. 2020), including distal symmetric polyneuropathy, characterized by numbness, tingling, pain, or weakness that begins in the feet and progresses proximally (Yovera-Aldana et al. 2021). DPN significantly reduces quality of life and predisposes individuals to falls, injuries, infections, and amputations (Feldman et al. 2019). Its prevalence is between 21.3% with 34.5% in T2DM, and 7% to 34.2% in type 1 DM (T1DM) (Yovera-Aldana et al. 2021). DPN is commonly categorized into symmetric polyneuropathies (with motor, sensory, and autonomic manifestations), focal, and multifocal neuropathies, and mixed forms (Thomas 1997). Its renewed classifications include typical DPN (mainly distal symmetric sensorimotor polyneuropathy) and atypical conditions (such as mononeuritis multiplex, and thoracic radiculopathy) (Tesfaye et al. 2010). In the theories of traditional Chinese medicine (TCM), DPN was classified into the following four distinct categories: cold coagulation blood stasis syndrome, blood deficiency cold coagulation syndrome, Qi deficiency blood stasis syndrome, and cold dampness spleen syndrome. (Tian et al. 2020).

Management of DPN typically involves glycemic control, lipid management, and pharmaceutical interventions for neuropathic pain (American Diabetes Association Professional Practice Committee 2024). Early glycemic control in T1DM can delay or prevent DPN (DCCT Research Group 1995; Albers et al. 2010), yet its effectiveness is less pronounced in T2DM (Callaghan et al. 2012). Dyslipidemia is a key factor in neuropathy development (Andersen et al. 2018), and lifestyle interventions such as weight loss and physical activity have shown positive effects in DPN management, rather than conventional lipid-lowering medications (Afshinnia et al. 2022). Pharmaceutical treatments such as gabapentinoids, sodium channel blockers, serotonin-norepinephrine reuptake inhibitors (SNRIs), and tricyclic antidepressants (TCAs) are recommended for neuropathic pain (Price et al. 2022), and combination therapy is often more effective than monotherapy (Tesfaye et al. 2022). However, optimal management of DPN remains a challenge, necessitating the exploration of innovative therapeutic approaches.

Emerging TCM-based treatments offer innovative and effective strategies for DPN management. TCM, with its long history in disease prevention and treatment, has garnered global attention. Recent studies have developed novel herbal products to address the growing prevalence of DPN (Hu M et al. 2023; Jiang et al. 2023; Liu Q et al. 2023; Wang Q et al. 2023; Zhang et al. 2024). Knowledge of the properties and scientific evidence supporting TCM’s role in DPN management is crucial. This review focused on the latest TCM approaches for DPN management, delving into contemporary research on TCM applications for DPN treatment.

## Methods

### Study selection

A literature review was conducted using PubMed, the Cochrane Neuromuscular Disease Group Specialized Register (CENTRAL) and the Chinese National Knowledge Infrastructure Database (CNKI), with TCM, DPN, and TCM treatment of DPN as keywords. Eventually, about 130 studies published before 2023 were included, focusing on preclinical studies and clinical trials assessing the effectiveness of TCM in treating DPN.

Inclusion criteria: (1) Clinical studies on treatment of diabetic peripheral neuropathy with traditional Chinese medicine; (2) Preclinical studies on treatment of diabetic peripheral neuropathy with traditional Chinese medicine; (3) The mechanism of treating diabetic peripheral neuropathy with traditional Chinese medicine.

Exclusion criteria: (1) External use of traditional Chinese medicine in treating diabetic peripheral neuropathy; (2) Treatment of diabetic peripheral neuropathy with the combination of traditional Chinese medicine.

### Classification of traditional Chinese medicine for DPN treatment

TCM is classified into three categories: single herbal extracts, herbal compound prescriptions, and Chinese patent medicines. The Mudan granule is the only drug currently marketed by the National Medical Products Administration for DPN, while most herbal extracts, compounds, and patent medicines remain in the preclinical or clinical study phases.

### Single herbal extracts

Based on their chemical structures, single herbal extracts in TCM are broadly categorized into glycosides, phenols, alkaloids, flavonoids, and other types. Despite the differences in their chemical composition, these extracts commonly target pathological indicators of DPN, such as the structure of the sciatic nerve, nerve conduction velocity (NCV), and nerve-related proteins. Only a few representative herbs are presented here; details of others are provided in Table 1.

**Table 1.** Single herbal extracts for the treatment of DPN.

Name	Extract source and Producer	Dose Range and Duration	Model	Control	Reference
Notoginsenoside R1	*Panax notoginseng,* brought from Sigma-Aldrich (St. Louis, MO)	10–100 μM for 48h, 50 μM was applied as optimum dosage	RSC96 cells	Vehicle	(Wang W et al. 2019)
Diosgenin	*Dioscorea nipponica Makino,* Solarbio Science & Technology Co., Ltd.,	50 and 100 mg/kg/day, i.g. for 8 weeks	Male C57 mice	Vehicle	(Leng et al. 2020)
Astragaloside IV	*Astragali radix,* Sigma-Aldrich, USA	Rats:20, 40 and 80 mg/kg/day, i.g. for 6 weeks Cells: 0,5,10,15 and 20μg/ ml for 48h	GK rats and RSC96 cells	Mecobalamin	(Yin et al. 2021)
Saikosaponin d	Radix Bupleuri, Chengdu Herbpurify Co., Ltd, Chengdu, China	1.0, 1.5 and 2.0 mg/kg/day, i.p. for 8 weeks	Male SD rats	Vehicle	(Xiang et al. 2023)
Salidroside	*Rhodiola rosea,* Tauto Biotech, Shanghai, China	50 and 100 mg/kg/day, i.g. for 6 weeks	Male SD rats	Vehicle	(Zheng et al. 2021)
Paeoniflorin	*Paeonia lactiflora* Pall. (Ranunculaceae), Liwah Pharmaceutical	Rats: 5.6 mg/kg/day for 12 weeks Cells: 10 μM for 24h	Male SD rats and RSC96 cells	Vehicle	(Yang X et al. 2022)
Gentiopicroside	*Gentiana lutea,* Nanjing Jingzhu Biological Science and Technology Co., Ltd., Nanjing, Jiangsu, China	50 and 100 mg/kg/day, i.g. for 2 weeks	Female SD rats	Oxcarbazepine	(Lu et al. 2018)
Curcumin	*Curcuma longa L.,* Dalian Meilun Biotech Co., Ltd., Dalian, China	50, 100 and 150 mg/kg/day, i.g. for 4 weeks	Male SD rats	Vehicle	(Zhang WX et al. 2022)
Magnolol	*Magnolia Officinalis* J&K Scientific Ltd., Shanghai, China	15 and 30 mg/kg/day, i.g. for 4 weeks	Male C57BL/6 mice and db/db mice	Vehicle	(Yang J et al. 2022)
Honokiol	*Magnolia Officinalis* Aladdin, Shanghai, China	Rats: 25, 50 and 100 mg/kg/day, i.g. for 4 weeks Cells: 2.5, 5 and 10 μM for 48h	Male SD rats and RSC96 cells	α-lipoic acid	(Hu M et al. 2023)
Salvianolic acid A	*Salvia miltiorrhiza* Bunge (Lamiaceae) China Academy of Medical Sciences Institute of Medicine	0.3 and 1 mg/kg/day, i.g. for 8 weeks	KK-4Y mice	Valsartan	(Xu 2020)
Salvianolic acid B	*Salvia miltiorrhiza* Bunge (Lamiaceae) Sigma-Aldrich, St. Louis, MO, USA	0.1, 1 and 10 μM for 72h	RSC96 cells	Vehicle	(Wang J et al. 2019)
Resveratrol	Natural plants and fruits	10 ml/kg 10% resveratrol, i.g. for 12 weeks	CD1/ICR mice	Vehicle	(Zhang et al. 2021)
Vincamine	*Madagascar periwinkle* Absin, Shanghai, China	30 mg/kg/day, i.g. for 4 weeks	Male C57BL/6J mice and db/db mice	Vehicle	(Xu JW et al. 2023)
Lycorine	*Lycoris radiate* MCE Co., Monmouth Junction, NJ, USA	Rats: 20 mg/kg/ two days, i.g. for 3 weeks Cells: 0.96, 1.44, 2.87 and 5.74 μg/ml for 48h, 2.87μg/ml was applied as optimum dosage	C57BL/6 male mice and RSC96 cells	Vehicle	(Yuan et al. 2022)
Berberine	*Rhizoma coptidis* Sigma Aldrich (St.Louis, MO, USA)	Rats: 50 and 100 mg/kg/day, i.g. for 2 weeks Cells: 5 and 10 μM for 24h	Male SD rats and N2A neuroblastoma cells	Vehicle	(Yerra et al. 2018)
Jatrorrhizine	*Rhizoma coptidis* from Good Agricultural Practices Demonstration Base in Shizhu City (Chongqing, China), extracted by ethanol for 24h 3 times and then collected and evaporated to dryness under rotary evaporators to obtain the ethanol extract	Rats: 50,100 and 200 mg/kg/day, i.g. for 8 weeks Cels:10, 20 and 40 mM for 24h	db/db mice and RSC96 cells	Vehicle	(Gong et al. 2023)
Puerarin	*Radix puerariae* Phytomarker Ltd (Tianjin, China)	1, 10 and 100 μM for 48h	Schwann cells from the sciatic nerves of SD rats	Vehicle	(Wu et al. 2012)
Quercetin	Fruits and vegetables	50 mg/kg/day, i.g. for 12 weeks	Male SD rats	Vehicle	(Xie et al. 2020b)
*Hedysarum* polysaccharide	*Hedysarum* Weikeqi Biological Technology Company (Chengdu, China)	Rats: 50, 100 and 200 mg/kg/day, i.g. for 8 weeks Cells: 30, 60, 120 and 240 mg/L for 48h	Male ob/ob mice and Schwann cells from the sciatic nerves of SD rats	α-lipoic acid	(He et al. 2022)
*Lycium barbarum* polysaccharide	*Lycium barbarum* Ningxia Agricultural and Forestry College	100 and 500 mg/kg/day, i.g. for 12 weeks	Male SD rats	α-lipoic acid	(Liu et al. 2018)
Muscone	*Musk* Selleck Chemicals (Houston, TX, USA)	0.1, 1, 10 and 50 μM for 24, 48 and 72h	RSC96 cells	Vehicle	(Dong et al. 2019)

#### Preclinical research - glycosides

Glycosides generally refer to compounds composed of aglycones and monosaccharides. This paper mainly introduced saponins. Saponins are a group of compounds widely distributed in the plant kingdom, characterized by their structure containing triterpenoid or steroid glycoside ligands and one or more sugar chains (Güçlü-Ustündağ and Mazza 2007). At present, glycosides are widely studied in metabolic syndrome, cardiovascular disease, neuropathy and cancer (Guo et al. 2023).

Panax notoginseng, derived from the root of *Panax notoginseng* (Burk.) F. H. Chen (Araliaceae), is a perennial herb. It is deemed to promote blood circulation and treat cardiovascular disease (Hao et al. 2017). Research studies have highlighted the benefits of active saponin components extracted from *Panax notoginseng* in treating various diseases, including diabetic retinopathy, cardiac lipotoxicity, and rheumatoid arthritis (Zhou et al. 2019; Jiao et al. 2021; Tian et al. 2022). In a study conducted by Wang W et al. (2019), RSC96 cells were cultured with notoginsenoside R1 (NGR1) at concentrations ranging from 10 ∼ 100 µM, followed by treatment with 100 mM glucose to simulate hyperglycemic conditions. The results showed that NGR1 treatment in RSC96 cells under high glucose conditions led to a reduction in reactive oxygen species (ROS) generation and an increase in the expression of nerve growth factor (NGF) and brain-derived neurotrophic factor (BDNF), compared to the high glucose group without treatment.

Diosgenin, a natural steroidal saponin extracted from *Dioscorea nipponica* Makino, *Dioscorea opposite* Thunb*.,* and *Dioscorea zingiberensis* Wright (Dioscoreaceae), has a variety of beneficial effects, including hypoglycemic (Xu et al. 2020), cardiovascular protective (Li et al. 2021), and hypolipidemic properties (Gong et al. 2010). Leng et al. (2020) showed that DPN mice received low (50 mg/kg) and high (100 mg/kg) doses of diosgenin through continuous gavage for 8 weeks. They concluded that diosgenin treatment led to an improvement in blood glucose levels and body weight in diabetic mice. Additionally, diosgenin significantly increased tail withdrawal latency, relieved mechanical hyperalgesia in diabetic animals and improved the pathological changes of the sciatic nerve. Similarly, it also reduced malondialdehyde (MDA) levels while enhancing the activities of superoxide dismutase (SOD) and glutathione peroxidase (Gpx).

Several other glycosides derived from TCM extracts may play therapeutic roles in treating DPN through various pharmacological mechanisms. These include astragaloside IV (AS-IV) (Ben et al. 2021; Yin et al. 2021), saikosaponin D (SSD) (Law et al. 2014; Zhou et al. 2021; Xiang et al. 2023; Xu X et al. 2023), salidroside (Li R et al. 2019; Xue et al. 2019; Zheng et al. 2021; Jin et al. 2022), paeoniflorin (Yang X et al. 2016; Yang X et al. 2022), gentiopicroside (Gent) (Lu et al. 2018; Xiao et al. 2020, 2022), and polydatin (Ji et al. 2012; Jiang et al. 2013; Gong et al. 2017).

#### Preclinical research - phenols

Phenols have different chemical structures and activities with more than 8,000 kinds which can be found in vegetables, seeds, fruits, nuts, red wine, tea, and many other food sources. In structure, phenols are metabolites of secondary plants, characterized by at least one aromatic ring connected with one or more hydroxyl groups (Eseberri et al. 2022). Phenols are widely used in disease research due to their outstanding anti-inflammatory and antioxidant effects (Azuma et al. 1986).

*Curcuma long*a L. (Zingiberaceae), with its long history of use as a traditional medicine in Asian countries, has attracted significant scientific interest (Memarzia et al. 2021). The main pharmacological component of *Curcuma longa* is curcumin. During the past decade, numerous studies have identified that curcumin has a wide range of biological effects, including antioxidant, neuroprotective, anti-inflammatory, and anticancer properties (Hussain et al. 2017; Kunnumakkara et al. 2017; Tanrıkulu-Küçük et al. 2019; Bhat et al. 2019). *In vitro* and *in vivo* studies have been conducted to investigate the efficacy of curcumin in treating peripheral neuropathies. It was noted that curcumin facilitates nerve regeneration and enhances NGF expression in rats with DPN (Ma et al. 2016; Zhang WX et al. 2022). Electron microscopy analyses have shown that in curcumin-treated groups, the sciatic nerve cross-sections are structurally intact, with improved segregation of myelinated nerve fibers. Meanwhile, both the mechanical withdrawal threshold (MWT) and NCV have been observed to increase proportionally with the duration and dosage of curcumin administration (Zhang WX et al. 2022).

The extracts of *Magnolia officinalis* Rehd. et Wils. (Magnoliaceae), specifically magnolol (MG) and honokiol (HNK), are lignin compounds recognized for their broad application value. Recent studies have demonstrated their anti-inflammatory, antioxidant, anticancer, and other pharmacological effects (Zhang and Tang 2012; Rauf et al. 2021). Both magnolol and honokiol have been proven to possess neuroprotective effects, with mechanisms related to their antioxidant properties and the antagonism of excitatory amino acids, making them potential therapeutic agents for neurodegenerative diseases (Lin et al. 2006).

In a study by Yang J et al. (2022), both T1DM and T2DM mice were used as models for DPN. They revealed that MG enhanced neurological functions in DPN mice by improving sensitivity to thermal and mechanical stimuli, and significantly increasing motor nerve conduction velocity (MNCV). In addition, MG was observed to enhance the density of intraepidermal nerve fibers (IENF) on the foot skin of DPN mice and to protect the myelin sheath structure of the sciatic nerve, as determined by immunofluorescence analysis. Similarly, honokiol, whose structure is similar to MG, exhibited comparable effects. Hu M et al. (2023) indicated honokiol improvements in sciatic NCV, increased thermal and mechanical sensitivity in diabetic rats, and relief from neuropathy symptoms. Moreover, honokiol mitigated high glucose-induced damage in Schwann cells.

Myelin basic protein (MBP) is essential for myelination and nerve health. Similarly, resveratrol, a compound widely distributed in plants, has also been identified to play a role in neuroprotection (Koushki et al. 2018; Nadile et al. 2022). Treatment of resveratrol in mice induced by streptozotocin revealed that resveratrol could enhance the expression of MBP to exact a protective effect on myelin (Zhang et al. 2021). Moreover, salvianolic acid A (Xu 2020) and salvianolic acid B (Wang QQ et al. 2019; Wang SX et al. 2010), derived from *Salvia miltiorrhiza* Bunge (Lamiaceae), were proved to improve DPN *in vivo* and *in vitro*.

#### Preclinical research - alkaloids

Alkaloids have a great influence on the lives of animals and human beings. However, their biosynthesis in plants involves many catalytic steps, and the diversification of structure is only associated with the appearance of nitrogen atoms in heterocyclic rings (Ziegler and Facchini 2008). At present, it is demonstrated that plant alkaloids not only have anticancer and antibacterial effects but also have the potential to ameliorate hypertension and neurological function (Bhambhani et al. 2021).

Vincamine, a monoterpene indole alkaloid extracted from *Madagascar periwinkle* (Apocynaceae), is clinically used to treat cerebrovascular diseases by increasing cerebral blood flow and oxygen consumption to improve cognitive dysfunction (Zhao et al. 2023). Researchers have hypothesized that vincamine could potentially aid in the treatment of DPN in mice. The proposed effects included improving blood flow velocity and perfusion in foot pads and sciatic nerve tissues, preventing myelin sheath damage in sciatic nerves, and ameliorating the density damage of intraepidermal nerve fibers (IENF) in foot skin (Xu JW et al. 2023).

Lycorine, an isoquinoline alkaloid extracted from the *Lycoris radiata* herb (Amaryllidaceae), exhibits multiple pharmacological effects, such as anticancer, anti-inflammation, and antioxidative stress (Roy et al. 2018; Wu et al. 2021). Studies involving *in vivo* and *in vitro* administration of lycorine have shown that it can reduce the thermal threshold by 36.12% and reverse MWT, demonstrating its potential effectiveness in treating neuropathy (*p* < 0.05, both) (Yuan et al. 2022).

*Rhizoma coptidis* refers to the dried rhizome of plants such as *Coptis chinensis* Franch., *Coptis deltoidea* C. Y. Cheng et Hsiao, or *Coptis teeta* Wall. (Ranunculaceae) (Wang J et al. 2019). The main active components extracted from these plants are berberine and jatrorrhizine (JAT), both isoquinoline alkaloids known to alleviate DPN. Berberine is used primarily in clinical settings for treating intestinal infections, due to its anti-inflammatory properties. Behavioral assays demonstrated increases in thresholds for mechanical allodynia and thermal hyperalgesia during berberine treatment, as measured by the von Frey test and the tail-flick test (Zan et al. 2017; Yerra et al. 2018). In addition, Gong et al. (2023) found that JAT offers more protection against DPN than other flavonoid alkaloids. After the treatment of JAT (50, 100 and 200 mg/kg) in db/db mice for eight consecutive weeks, it significantly improved the general physiological functions and nerve morphology. Treatment significantly promoted myelin formation by improving sciatic nerve conduction velocity, increasing heat and pain sensitivity, and elevating myelin protein levels.

#### Preclinical research - flavonoids

It’s well known that flavonoids are the most abundant and widely distributed secondary plant metabolites in fruits and vegetables (Čižmárová et al. 2023). They have various pharmacological properties like antibacterial, antiviral, antitumor, anti-inflammatory, antiallergic, vasodilation, and cardioprotective effects (Chiorcea-Paquim 2023).

*Pueraria lobata*, officially recognized in the Chinese pharmacopeia and known for its benefits to muscles, brain, liver, and kidneys, is the dried root of *Pueraria lobata* (Willd.) Ohwi (Fabaceae) or *Pueraria thomsonii* Benth (Fabaceae) (Wang et al. 2020; Li et al. 2023). Puerarin, the primary isoflavonoid bioactive ingredient isolated from *Pueraria lobata*, has been widely used in treating cardiovascular diseases, diabetes, diabetic complications, and neurodegenerative disorders (Zhou et al. 2014). *In vivo,* studies verified that puerarin can enhance the activity of SOD in sciatic nerve tissue and helps reduce oxidative stress-related damage, inhibit excessive free radical generation, improve metabolic status, and consequently repair damaged nerve fibers. Additionally, puerarin has been shown to increase nerve conduction velocity and improve neurosensory symptoms (Wu et al. 2012).

Quercetin, another significant flavonoid abundantly found in fruits and vegetables, is known for its anti-inflammatory, anticancer, neuroprotective, and anti-hyperglycemic properties (Hosseini et al. 2021). It’s proved that quercetin can mitigate high glucose-induced damage to Schwann cells by modulating autophagy (Qu et al. 2014). Xie et al. (2020b) identified that quercetin improves peripheral nerve function by alleviating the imbalance of the gut microbiota associated with DPN phenotypes. In the research conducted by Zhao et al. (2021), DPN rats were treated daily with low (30 mg/kg) and high (60 mg/kg) doses of quercetin for six weeks. Subsequent observations of pathomorphological changes in sciatic nerves revealed that nerve fibers in DPN rats were loosely arranged, disordered, less intensely stained, and varied in size. Further investigation revealed that quercetin significantly reduced the levels of tumor necrosis factor-α (TNF-α) (*p* < 0.05) and interleukin-1β (IL-1β) (*p* < 0.001) which were negatively correlated with MWT and NCV. Overall, this study suggested that quercetin could contribute to improving nerve function by reducing inflammatory factors.

#### Preclinical research - others

A substantial number of studies have demonstrated that polysaccharides found in certain Chinese medicinal herbs, such as *Lycium barbarum* polysaccharide and *Hedysarum* polysaccharide, can effectively improve DPN. Both *Lycium barbarum* polysaccharide and *Hedysarum* polysaccharide not only reduced blood glucose levels but also alleviated symptoms of allodynia and hyperalgesia. Furthermore, they helped preserve NCV and mitigate structural damage to nerve fibers (Liu et al. 2018; He et al. 2022). Additionally, researchers (Dong et al. 2019) discovered that decreased cell viability and increased apoptosis in RSC96 cells, induced by high glucose levels, could be reversed by applying muscone. However, the extent of muscone effectiveness on DPN still requires confirmation through further *in vivo* experiments.

#### Clinical research

Although most studies on herbal extract monomers, such as those derived from *Panax notoginseng*, curcumin, and puerarin, have been conducted *in vitro* and *in vivo*, there are fewer clinical trials. Moreover, products like the *Panax notoginsen* saponins capsule and puerarin injection have shown promising clinical efficacy in DPN patients. A clinical investigation involving 50 cases demonstrated that the *Panax notoginsen* saponin capsule effectively improved total bilirubin levels (*p* < 0.05) and vibrating perception threshold (VPT) (*p* < 0.05). While there were no significant differences in serum iron levels (*p* > 0.05) between the treatment and control groups, the levels of hepcidin and ferritin in the treatment group [17.87 ± 5.27 ng/mL, 224.24 ± 207.44 ng/mL] were significantly lower than those of the mecobalamin group [23.06 ± 5.97 ng/mL, 422.19 ± 296.31 ng/mL] (Sun and Liu 2020).

Numerous studies have indicated that the hydrophobic nature of curcumin leads to poor absorption and rapid metabolism. To address this, nano-curcumin, known for its greater bioavailability, was used in a double-blind, randomized, parallel, placebo-controlled clinical trial (Asadi et al. 2019). The trial included 80 patients with diabetic sensorimotor polyneuropathy, aged 30 ∼ 60 years. Participants in the intervention group were instructed to take an 80 mg capsule of nano-curcumin after a meal for 8 weeks. Comparing the placebo and curcumin groups revealed that curcumin effectively reduced fasting blood sugar (FBG, *p* = 0.004), glycated hemoglobin (HbA1c, *p* < 0.001), the total neuropathy score (*p* < 0.001), and total reflex score (*p* = 0.04). Moreover, the reduction in FBG and HbA1c was correlated with a decrease in the severity of diabetic sensorimotor polyneuropathy (DSPN) (FBG: *p* = 0.02, *r* = 0.26; HbA1c: *p* = 0.04, *r* = 0.22). While the study showed that nano-curcumin supplementation could mitigate the severity of DSPN, the lack of male participants and the use of a single dose suggest the need for more comprehensive clinical studies in the future.

Puerarin injection, initially launched in China in 1993 as an adjunctive treatment for coronary heart disease, angina pectoris, and myocardial infarction, has also reflected its efficacy in treating DPN (Dong 2015; Yang 2016; Zhang 2017; Wang et al. 2018; Zeng 2020). In clinical studies comparing puerarin treatment with conventional therapies which receiving hypoglycemic therapy, α-lipoic acid, or vitamin B supplementation only, the observation group was administered an intravenous infusion of puerarin daily, with their general condition, symptom scores, and NCV closely monitored. The results indicated several significant findings: 1) the total effective rate of treatment in the observation group (91.67%) was markedly higher than in the control group (81.25%, *p* < 0.05), 2) patients receiving puerarin injection showed better improvement in symptom scores compared to before treatment (*p* < 0.05), 3) treatment positively influenced blood rheology (*p* < 0.05), enhancing nerve blood supply and reduced nerve damage, and 4) electromyogram (EMG) analysis revealed significant improvements in the motor nerve conduction velocity (MNCV) and sensory nerve conduction velocity (SNCV) of the median and common peroneal nerves after treatment (*p* < 0.05) (Wang et al. 2018).

### Self-composed Chinese herbal compound prescription

Chinese herbal compounding, a traditional therapeutic method with a rich history in China, has received increasing attention in recent years, particularly for treating complex and multifaceted diseases such as DPN. The preclinical and clinical studies were reviewed.

#### Preclinical research

Preclinical studies have demonstrated improvements in DPN with the combination of herbal medicines, including Tang-luo-ning (TLN), Jinmaitong, Jiaweibugan decoction, Huangqi Guizhi Wuwu decoction, Modified Huangqi Guizhi Wuwu decoction, Danggui Sini decoction, Mongolian prescriptions, Long-Qi decoction, and the Tangtong formula. The details are shown in Table 2.

**Table 2.** TCM compound formula for the treatment of DPN(Preclinical).

Name	Recipe Composition	Extraction method	Dosage/Daily	Model	Effects	Reference
Tang-luo-ning	*Astragali radix, Rehmannia glutinosa* Libosch*., Angelica sinensis*(Oliv.)Diels*, Lycium barbarum* L.*, Achyranthes bidentata* Bl*., Chaenomeles speciosa(Sweet)*Nakai*, Dipsacus asper* Wall. ex Henry*, Codonopsis pilosula*(Franch.)Nannf.	Prepare powder and dissolve the powder in distilled water	10.9 crude drug/kg/day and 21.8 crude drug/kg/day for 12 weeks	Male Sprague-Dawley (SD) rats	Oxidative and ER stress↓ Caspase-3, Bax↓	(Yang et al. 2017)
Mongolian prescriptions BAIMAI-SAN	*picroside II, verbascose, taurine, ellagic acid, Borneolum syntheticum*	/	0.1,0.3,0.9 mg/kg/day, i.g. for 75 days	Wistar rats	MNCV, SNCV↑	(Liu et al. 2011)
Jinmaitong	*Cuscuta chinensis Lam., Ligustrum lucidum Ait., Herba Ecliptae, Prunella vulgaris* L., *Litchi chinensis* Sonn*., Cinnamomum cassia Presl, Corydalis yanhusuo* W.T.Wang*, Persicae semen, Cassiae semen, Asari radix et rhizome, Buthus martensii Karsch,* Hirudo nipponica Whitman	After grounding into powder and mixing, per 100 mg powder is extracted in 1 mL of water and 1 mL of methanol.	0.4375 g/kg/day, 0.875 g/kg/day, and 1.75 g/kg/day for 8 weeks, 12 weeks or 16 weeks.	Male SD rats	①IGF-1, IGF-1R, gene P0 and PMP22↑ Active Wnt/β-catenin pathway	①(Song et al. 2019) ②(Song et al. 2021)
Jiaweibugan decoction	*Lycium barbarum* L.,* Chaenomeles speciosa (Sweet)*Nakai*, Angelica sinensis (*Oliv.) Diels*, Ligusticum chuanxiong* *Hort*.*, Rehmanniae radix praeparata, Paeonia lactiflora* Pall.,* Taxillus chinensis (DC.)* Danser*, Ophiopogon japonicus*(L.f)Ker-Gawl*., Trichosanthis radix*	Decoct with 1 200 mL water 30 min, warm on low heat twice 30 min.	28.6 g/kg per day for 8 weeks.	Male Wistar rats	MDA, NF-kB p65 mRNA and p38 mitogen-activated protein kinase ↓; GSH↑	(Wang et al. 2013)
Huangqi Guizhi Wuwu decoction	*Astragali radix, Cinnamomum cassia Presl*, *Paeonia lactiflora* Pall.*, Zingiber officinale Rosc., Ziziphus jujuba Mill.*	/	①10% Medicated serum ②3.5 g/kg, 7 g/kg and 14 g/kg for 8 weeks	①RSC96 cells ②*db/db* mice	①ROS↓;T-SOD↑ ②TNF-α, IL-1β and IL-6, MDA↓ IL-10, GSH, T-SOD and NGF↑	①(Gao 2022) ②(Zhang et al. 2024)
Modified Huangqi Guizhi Wuwu Decoction	*Astragali radix, Cinnamomum cassia Presl, Paeonia lactiflora Pall., Ligustrum lucidum Ait., Corydalis yanhusuo* W.T.Wang,* Coptis chinensis* Franch.*, Spatholobus suberectus* Dunn*,* Hirudo nipponica Whitman	Chinese medicinal granules are prepared with distilled water.	1.25g/kg/d and 2.5g/kg/d	Male SD rats	①Bax, Caspase-12↓ ②ROS, p-IRE1α and CHOP↓	①(Zhang et al. 2023a) ②(Zhang et al. 2023b)
Danggui Sini Decoction	*Angelica sinensis* (Oliv.) Diels*, Cinnamomum cassia Presl, Asari radix et rhizome, Paeonia lactiflora* Pall*., Tetrapanax papyrifer(*Hook.)K.Koch*, Ziziphus jujuba* *Mill*.,* Glycyrrhizae radix et rhizoma*	Decocting	1.84 g/kg/d and 7.36 g/kg/d for 8 weeks	Wistar rats	MNCV↑; NF-κB↓	(Cheng et al. 2019)
Long-Qi Decoction	*Cinnamomum cassia Presl, Morus alba* L.,* Angelica sinensis*(Oliv.)Diels,* Astragali radix, Paeoniae radix rubra, Pheretima, Spatholobus suberectus* Dunn	Heat and reflux for 2h, add ethanol, take the supernatant and concentrate it, then grind it into power.	1.58g/kg,3.17g/kg and 6.33g/kg,	Male Wistar rats	NCV↑; Sorbitol and aldose reductase↓	(He et al. 2019)
Tangtong Formula	*Astragali radix, Cinnamomum cassia* Presl,* Paeonia lactiflora* Pall.,* Asari radix et rhizome, Eupolyphaga steleophaga, Curcuma longa* L.,* Ligusticum chuanxiong* *Hort*.	Decocting	9.17g/kg/d, 18.33g/kg/d and 36.67 g/kg/d	SD rats	MNCV, SNCV↑	①(Wei et al. 2019) ②(He 2021)
Yiqi Huoxue Tongluo Formula	*Astragali radix, Angelica sinensis(*Oliv.)Diels,* Rehmannia glutinosa* Libosch*., Paeonia lactiflora* Pall.*, Persicae semen, Carthamus tinctorius* L.,* Achyranthes bidentata* Bl.,* Paeoniae radix rubra, Spatholobus suberectus* Dunn*, Chaenomeles speciosa(Sweet)*Nakai*, Caesalpinia sappan* L.,* Corydalis yanhusuo* W.T.Wang*, Glycyrrhizae radix et rhizoma praeparata cum melle*	Soaking, decocting, filtering, and concentrating	1.84 g/kg/d and 7.36 g/kg/d for 8 weeks	Wistar rats	MNCV↑; NF-κB↓	(Zhang L et al. 2022)
Tang Bi Kang (TBK) granule	*Astragali radix, Ligustrum lucidum Ait., Cinnamomum cassia Presl, Paeonia lactiflora* Pall.,* Spatholobus suberectus* Dunn*, Scutellaria baicalensis Georgi, Coptis chinensis Franch.,* Hirudo nipponica Whitman,* Corydalis yanhusuo* W.T.Wang	Soak 1h, decoct thrice for 2h each, add alcohol to concentrate, reflux-extracted thrice for 1h each.	4.28g/kg, 8.56g/kg and 17.12g/kg for 4 weeks	Wistar rats	FBG, TNF-αand IL-6, MDA↓ MNCV, SNCV, SOD and GSH-pX↑	①(Yang, XW et al. 2015) ②(Liu G et al. 2023)
Compound XiongShao Capsule	*Paeonia lactiflora Pall., Cyathula officinalis Kuan, Ligusticum chuanxiong Hort., litchi rind, Saposhnikovia divaricate (Turcz.)*Schischk*., Cinnamomum cassia Presl, Sargassum, Polygonatum sibiricum Red., Astragali radix, Morus alba* L.*, Silybum marianum(*L*.)*Gaertn.*, Orostachys fimbriata(Turcz.)*Berg*.*	Decoct first for 2h with distilled water eight times and then for 1h with distilled water six times.	0.12 g/kg, 0.36 g/kg and 1.2 g/kg for 8 weeks	Male SD rats	NCV, SOD↑; Bax, caspase-3, AGEs and NOS↓	(Yu et al. 2021)
Compound Qiying Granule	*Astragali radix, Polygonatum sibiricum* Red.,* Salvia miltiorrhiza* Bge.*, Cryptotympana pustulata Fabricius, chickpea*	/	1.17 g/kg/d, 2.34 g/kg/d and 4.68 g/kg/d for 4 weeks	Male Wistar rats	IL-6, IL-1β, TNF-α and ROS↓	(Hu Y et al. 2023)
Liuweiluobi granule	*Hippophae rhamnoides* L.,* Carthamus tinctorius* L.,* Lonicerae japonicae Flos, Scutellaria barbata* D.Don*, Achyranthes bidentata* Bl.,* Ephedra sinica Stapf*	/	4.4g/kg, 8.7g/kg and 17.4g/kg for 12 weeks	Male db/db mice	FGB↓ MNCV, SNCV↑	(Liu 2021)
Chaihu Shugan Power	*Bupleurum chinense DC., Citrus reticulata Blanco, Paeonia lactiflora Pall., Citrus aurantium* L.*, Ligusticum chuanxiong Hort., Cyperus rotundus* L.*, Glycyrrhizae radix et rhizoma praeparata cum melle*	Soak 2 h, decoct twice, mix and filter, and concentrate by water bath evaporation to obtain concentrated medicine.	3.15g/kg,6.30g/kg and 12.60 g/kg for 8 weeks	Male SD rats	MNCV, TRPV1, CGRP, TNF-α and IL-1β↑	(Wang Y et al. 2023)
QiGui TangTongNing Granule	*Astragali radix, Angelica sinensis (*Oliv.) Diels,* Rehmannia glutinosa* Libosch*., Pueraria lobata(Willd.)Ohwi, Corydalis yanhusuo* W.T.Wang*, Spatholobus suberectus* Dunn*, Clematis chinensis Osbeck*	/	1.575g/kg/d, 3.15 g/kg/d and 6.30 g/kg/d for 12 weeks	Male SD rats	NCV, PWMT↑ AGEs↓	(Liu 2020)

Tang-luo-ning (TLN) is a traditional Chinese herbal compound prescription consisting of eight herbs. Both *in vivo* and *in vitro* studies demonstrated that TLN downregulates the expression of apoptosis-related proteins, such as the caspase-3 and Bcl-2-associated X protein (Bax). It inhibits sciatic nerve apoptosis, improves motor and sensory nerve conductance, and enhances thermal sensory threshold in DPN rats. Additionally, TLN attenuates demyelination and axonal atrophy (Yang et al. 2017). In DPN rats, TLN showed improvement in the thermal perception threshold (TPT) of the rat tail, NCV, and demyelination, and reversed the expression levels of apoptosis-related proteins (Li X et al. 2019).

Jinmaitong (JMT), consisting of 12 drugs, is a prescription from Peking Union Medical College Hospital of the China Academy of Medical Sciences. It has been reported to inhibit inflammatory reactions, modulate the gut microbiota, and affect neuregulin 1 in DPN rats (Sun et al. 2019; Xie et al. 2020a). JMT improves NCV and pain and temperature sensation in rats. Pharmacological studies suggest that its potential mechanism involves repairing nerve damage and enhancing nerve regeneration. This is achieved by increasing the content of insulin-like growth factor 1 (IGF-1), insulin-like growth factor 1 receptor (IGF-1R), myelin protein zero (MPZ), and peripheral myelin protein 22 (PMP22) expression, as well as related mRNAs in tissues under high glucose conditions (Song et al. 2019, 2021).

Jiaweibugan decoction, which primarily contains ingredients from nine well-known traditional Chinese herbs, exhibits significant effects on DPN through antioxidative stress. Research has verified that Jiaweibugan decoction markedly decreases serum MDA level (*p* < 0.05) and increases the glutathione (GSH) level (*p* < 0.05) compared to the model group (Wang et al. 2013).

Huangqi Guizhi Wuwu decoction (HGW), comprising five traditional medicines of the ‘Synopsis of Golden Chamber’ also demonstrates efficacy in treating DPN by inhibiting oxidative stress. Under hyperglycemic conditions, HGW treatment in RSC96 cells has been shown to reduce the level of ROS while upregulating the activity of total superoxide dismutase (T-SOD) (Gao 2022). It’s revealed that HGW ameliorates the typical phenotypes of DPN in mice by changing the gut microbiota and plasma metabolism, which include an increase in tactile perception levels and thermal latency, as well as a decrease in SNCV and MNCV (Zhang et al. 2024).

Similarly, the modified Huangqi Guizhi Wuwu decoction (MHGW) has been the subject of study. Lucas Fast Blue staining (LFB) of sciatic nerve samples from rats in the administration group indicated more regular myelin sheath morphology, less swelling of the myelin sheath, more orderly nerve fibers, and a more uniform density (Zhang et al. 2023a, 2023b).

In addition to decoctions, various other dosage forms of proprietary Chinese medicines, such as Tang Bi Kang granule (TBK), Compound Qiying granule, QiGui TangTongNing granule, Liuweiluobi granule, Chaihu Shugan powder, and Compound XiongShao capsule (CXSC), have been developed in hospitals or research institutes for DPN treatment.

The Tang Bi Kang granule, based on the Huangqi Guizhi Wuwu decoction, is an empirical formula developed by Professor Liu Tonghua of Beijing University of Chinese Medicine. It has shown significant efficacy in improving DPN. Studies on TBK (Yang XW et al. 2015; Liu G et al. 2023) indicate that it can significantly reverse MNCV and SNCV deficits and reduce axonal atrophy and demyelination. TBK achieves this by markedly decreasing FBG, inflammatory cytokines, and oxidative stress-related indices, as well as by lowering thermal perception thresholds.

Similarly, the Compound XiongShao capsule (CXSC) (Yu et al. 2021), derived from the traditional classical herb formula Buyang Huanwu Decoction, comprises 12 traditional Chinese herbs. A study on CXSC confirmed its neuroprotective effects in DPN rats by maintaining the integrity of myelin and axonal structures, and alleviating NCV impairments (MNCV: *F_5,36_* = 7.644, *p* < 0.0001; SNCV: *F_5,36_* = 12.83, *p* < 0.0001). Furthermore, CXSC was also effective in reducing mechanical hyperalgesia (*F_5,36_* = 18.24, *p* < 0.0001) and thermal hyperalgesia (*F_5,36_* = 8.45, *p* < 0.0001).

#### Clinical research

There is growing clinical interest in the use of empirical compound formulas for treating DPN. These include Xiaoketongbi formula (XF), Danggui Sini decoction and its modifications, Fuyang Tongluo decoction, modified Huangqi Guizhi Wuwu decoction, Tangmoning decoction, Buyang Huanwu decoction, and Fuyuan Huoxue decoction (Table 3).

**Table 3.** TCM compound formula for the treatment of DPN(clinical).

Name	Recipe Composition	Extraction method	Manufacturer	Dosage	Duration	Reference
Danggui Sini Decoction	*Angelica sinensis(Oliv.)Diels, Spatholobus suberectus* Dunn,* Pheretima, Glycyrrhizae radix et rhizoma praeparata cum melle, Asari radix et rhizoma, Paeonia lactiflora Pall., Astragali radix, Tetrapanax papyrifer(*Hook.)K.Koch*, Ziziphus jujuba Mill.*	Soak 10 min, decoct with 400ml water 20 min to keep 200ml, add 300ml water to decoct again and keep 150 ml medicinal juice, mix the juice and decoct.	The Research Department	350ml/d	4 weeks	(Cao 2018)
Modified Danggui Sini Decoction	*Astragali radix praeparata cum melle, Paeonia lactiflora Pall., Spatholobus suberectus* Dunn*, Angelica sinensis* (Oliv.) Diels,* Cinnamomum cassia Presl, Pheretima, Asari radix et rhizoma, Tetrapanax papyrifer*(Hook.)K.Koch,* Glycyrrhizae radix et rhizoma, Ziziphus jujuba Mill., Scolopendra subspinipes mutilans* L.Koch	Decoct with 300ml water.	The Research Department	300ml/d	4 weeks	(Yang 2017a)
Modified Danggui Sini Decoction	*Angelica sinensis* (Oliv.) Diels,* Paeonia lactiflora Pall., Asari radix et rhizoma, Cinnamomum cassia Presl, Tetrapanax papyrifer*(Hook.)K.Koch,* Ziziphus jujuba Mill., Glycyrrhizae radix et rhizoma praeparata cum melle*	Decocting	The Research Department	1 dose/d	4 weeks	(Chen 2018)
Xiaoketongbi Formula	*Persicae semen, Rheum officinale Baill., Astragali radix, Angelica sinensis* (Oliv.) Diels,* Salvia miltiorrhiza Bge.*	Extracted with hot water, concentrated, spray‐dried, processed into granules	Huarun Sanjiu Pharmaceuticals Co Ltd.	15 g/d for 10 weeks	10 weeks	(Lu et al. 2022)
Fuyan Tongluo Decoction	*Aconitum carmichaelii* Debx.,* Lonicera japonica Thunb., Atractylodes macrocephala Koidz., Asari radix et rhizome, Angelica sinensis* (Oliv.) Diels,* Glycyrrhizae radix et rhizoma praeparata cum melle*	Decocting	Shanxi Provincial Integrated TCM And WM Hospital	150ml/twice/d	8 weeks	(Zhang et al. 2019b)
Modified Huangqi Guizhi Wuwu Decoction	*Astragali radix, Paeonia lactiflora Pall., Pueraria lobata (Willd.)* Ohwi*, Salvia miltiorrhiza Bge., Cinnamomum cassia Presl, Ligusticum chuanxiong Hort., Angelica sinensis*(Oliv.)Diels	Soak 20-30 min, boil and then decoct with slow fire for 20-30 min to keep 200-300 ml juice.	The Research Department	200∼300 mL/d	/	(Cheng et al. 2020)
Tangmoning Decoction	*Astragali radix, Rehmannia glutinosa* Libosch*., Angelica sinensis* (Oliv.) Diels*, Ligusticum chuanxiong Hort., Cinnamomum cassia Presl, Cornus officinalis Sieb.et Zucc., Persicae semen, Pheretima, Paeoniae radix rubra, Achyranthes bidentata* Bl.,* Spatholobus suberectus* Dunn,* Salvia miltiorrhiza Bge., Glycyrrhizae radix et rhizoma, Lycium barbarum* L.,* Panax ginseng* C.A.Mey*., Corydalis yanhusuo* W.T.Wang*, Clematis chinensis Osbeck*	Decoct to keep 400ml juice.	The Research Department	400ml/d	A month	①(Chen and Zhang 2023) ②(Zhang 2020)
Huangqi Danshen Tongluo formula	*Astragali radix, Salvia miltiorrhiza Bge., Cinnamomum cassia Presl, Angelica sinensis* (Oliv.)Diels,* Spatholobus suberectus* Dunn*, Pheretima*	Decoct in water for 2 times/20 min and leave 500 ml juice.	The Research Department	500ml/d	4 weeks	(Shi 2019)
Buyang Huanwu Decoction	*Astragali radix, Cinnamomum cassia Presl, Achyranthes bidentata Bl., Eupolyphaga steleophaga, Angelica sinensis*(Oliv.)Diels,* Ligusticum chuanxiong Hort., Salvia miltiorrhiza Bge., Paeonia lactiflora Pall., Persicae semen, Carthamus tinctorius* L.,* Curcuma longa* L.,* Glycyrrhizae radix et rhizome, Ziziphus jujuba Mill.*	Decoct in water 2 times and mix the juice.	The Research Department	Twice/d	4 weeks	(Li 2021)
Yiqi Huoxue Tongluo formula	*Astragali radix, Dendrobll caulis, Scrophularia ningpoensis Hemsl., Spatholobus suberectus* Dunn*, Cinnamomum cassia Presl, Angelica sinensis* (Oliv.)Diels*, Paeonia lactiflora Pall., Pheretima, Clematis chinensis Osbeck, Cyathula officinalis Kuan, Glycyrrhizae radix et rhizoma*	Boil with strong fire, then decoct with slow fire.	The Research Department	1 dose/d	30 days	(Zhou 2019)
Qiteng Tongluo Yin	*Astragali radix, Salvia miltiorrhiza Bge., Cinnamomum cassia Presl, Angelica sinensis* (Oliv.) Diels*, Spatholobus suberectus* Dunn*, Pheretima*	Decoct to keep 300ml.	The Research Department	300 mL/d	12 weeks	(Yang et al. 2019)
Fuyuan Huoxue Decoction	*Guaweigen, Rheum officinale Baill., Angelica sinensis* (Oliv.) Diels*, Glycyrrhizae radix et rhizome, Bupleurum chinense DC., Persicae semen, Carthamus tinctorius* L.*, Pangolin Scales*	Decoct to keep 300ml.	The Research Department	300ml/d for 2 or 4 weeks	2 or 4 weeks	①(Zhang and Zhang 2018) ②(Zhang 2018)
Wenyang Huoxue Tongluo recipe	*Astragali radix, Spatholobus suberectus* Dunn,* Rehmanniae radix praeparata, Ligusticum chuanxiong Hort., Cyathula officinalis Kuan, Paeoniae radix rubra, Psoralea corylifolia* L.,* Carthamus tinctorius* L.,* Paeonia lactiflora Pall., Curcuma rhizome, Cinnamomum cassia Presl, Cinnamomum cassia Presl, Asari radix et rhizoma*	Decocting	The Research Department	1 dose/d	/	(Zhou 2017)
Yiqi Huoxue Decoction	*Astragali radix, Trichosanthis radix, Atractylodis rhizome, Salvia miltiorrhiza Bge., Angelica sinensis* (Oliv.) Diels,* Dioscorea opposita Thunb., Spatholobus suberectus* Dunn*, Bombyx batryticatus, Rehmanniae radix praeparata, Pheretima, Morus alba* L*.*	Decocting	The Research Department	1 dose/d	2 month	(Yang 2017b)
Qidan Dihuang Decoction	*Astragali radix, Salvia miltiorrhiza Bge., Rehmannia glutinosa* Libosch*., Fruit of Cornus officinalis Sieb.rt Zucc., Dioscorea opposita Thunb., Alisma plantago-aquatica Linn., Tree Peony Bark, Poria cocos (Schw.) Wolf, Spatholobus suberectus* Dunn,* Cinnamomum cassia Presl, Dendrobll caulis, Cyathula officinalis Kuan, Panax notoginseng*(Burk.)F.H.Chen	Decocting	The Research Department	1 dose/d	2 month	(Wang and Lu 2017)
Modified Taohong Siwu Decoction	*Persicae semen, Carthamus tinctorius* L.*, Ligusticum chuanxiong Hort., Rehmanniae radix praeparata, Paeonia lactiflora Pall., Angelica sinensis* (Oliv.) Diels*, Codonopsis pilosula (Franch.)Nannf., Astragali radix*	Decoct to keep 150ml juice.	The Research Department	300ml/d	2 month	(Peng et al. 2023)
Tangshen’an decoction	*Astragali radix, Morus alba* L.,* Pueraria lobata (Willd.)* Ohwi*, Salvia miltiorrhiza Bge., Achyranthes bidentata Bl., Dung of Trogopterus xanthipes Milne-Edwards, Olibanum, Cinnamomum cassia Presl, Commiphora myrrha Engl., Zaocys dhumnades(Cantor), huaPanax notoginseng(Burk.)*F.H.Chen,* Buthus martensii Karsch*	Decoct with 450ml water.	The Research Department	450mL/d	1 month	(Wu 2021)
Jiangtang Tongmailing Capsule	*Panax quinquefolium* L.,* Astragali radix, Cornus officinalis Sieb.et Zucc., Lycium barbarum* L.*, Ligusticum chuanxiong Hort., Angelica sinensis (*Oliv.) Diels*, Salvia miltiorrhiza Bge., Paeonia lactiflora Pall., Polygonatum sibiricum Red., Pangolin Scales, Achyranthes bidentata Bl., Morus alba L., Cinnamomum cassia Presl*	/	The Research Department	15capsules/d	2 month	(Mu 2016)
Danshen and Gergen granule	*Pueraria lobata (Willd.)* Ohwi,* Salvia miltiorrhiza Bge.*	/	The Research Department	1 dose/d	2 month	(Wen 2017)
Compound Xiongshao Capsule	*Paeonia lactiflora Pall., Cyathula officinalis Kuan, Ligusticum chuanxiong Hort., litchi rind, Saposhnikovia divaricate (Turcz.) Schischk., Cinnamomum cassia Presl, Sargassum, Polygonatum sibiricum Red., Astragali radix, Morus alba L., Silybum marianum(L.)Gaertn., Orostachys fimbriata(Turcz.)*Berg.	/	Shanghai fangxin pharmacy technology co., ltd	0.9 g, tid	6 month	(Cui et al. 2018)
Jiangtang Huoluo pill	*Panax ginseng* C.A.Mey*, Ophiopogon japonicus(L.f)Ker-Gawl., Schisandra chinensis(Turcz.)Baill, Trichosanthis radix, Dioscorea nipponica Makino., Angelica sinensis*(Oliv.)Diels*, Paeonia suffruticosa Andr., Scrophularia ningpoensis Hemsl., Salvia miltiorrhiza Bge., Morus alba* L.,* Zaocys dhumnades(Cantor), Pheretima, Scolopendra subspinipes mutilans* L.Koch,* Gallus gallus domesticus Brisson*	/	The Research Department	9g, tid	4 weeks	(Wu et al. 2019)

The Xiaoketongbi formula (XF), derived from the Tao-He-Cheng-Qi decoction in the ‘Treatise on Typhoid Fever’, primarily comprises Persicae semen, *Rheum officinale* Baill. (Polygonaceae), and *Astragali radix*. To assess its effectiveness in treating painful diabetic neuropathy, a single-center, randomized, single-blind, double-dummy, and parallel-controlled clinical trial involving 68 patients was conducted. After 10 weeks of treatment, the Brief Pain Inventory for Diabetic Peripheral Neuropathy (BPI-DPN) scores showed a significant decrease in both the XF and pregabalin groups, dropping from 42.44 ± 17.56 to 26.47 ± 22.22 and from 52.03 ± 14.30 to 37.85 ± 17.23 points, respectively (*p* < 0.001). However, there were no significant differences in the BPI-DPN score reduction between the two groups. Compared to the pregabalin group, the XF group demonstrated a significantly higher number of patients showing improvement [35.3% (12/34) vs. 11.8% (4/34), *p* = 0.045] and a more substantial absolute change in MNCV of the right median nerve (0.7 ± 2.3 in the XF group vs. −2.2 ± 4.1 in the pregabalin group, *p* = 0.004). No serious adverse effects were reported during the trial indicating the safety of XF (Lu et al. 2022).

Danggui Sini decoction (DGS), also originating from the ‘Treatise on Typhoid Fever,’ consists primarily of seven Chinese herbs. Clinical studies have demonstrated the efficacy of DGS and its modifications in treating DPN patients with symptoms of cold coagulation blood stasis syndrome, blood deficiency cold coagulation syndrome, *qi* deficiency blood stasis syndrome, and cold dampness spleen syndrome. Cao (2018) showed that DPN patients characterized by cold coagulation and blood stasis were selected as the observation group and treated with 350 mL/day of DGS juice for four weeks. The results indicated a higher total effective rate in the observation group compared to the control group. Additionally, there was a more significant improvement in MNCV and SNCV of the common peroneal and tibial nerves in the limbs of the observation group.

Furthermore, treating elderly diabetic peripheral neuropathy of the blood deficiency and cold coagulation type with the modified Danggui Sini decoction (MDGS) not only improved MNCV and SNCV, but also enhanced hemorheological indexes, reduced platelet aggregation, and promoted blood circulation after four weeks of treatment (Yang 2017a). Combining MDGS with acupressure was found to improve clinical symptoms and MNCV in treating DPN with *qi* deficiency and blood stasis (Yu 2016). Chen (2018) reported that improving renal function and hemorheology could alleviate the clinical symptoms of DPN patients with cold dampness and spleen trapping syndrome.

Fuyang Tongluo decoction (FYTL), a formula comprising six Chinese herbs based on DGS and modified Baizhu Fuzi Tang, was administered to 60 patients diagnosed with DPN and cold congealed blood stasis syndrome (Zhang Dong, Feng, Cui et al. 2019; Zhang, Dong, Feng, Guo 2019). It was revealed that FYTL significantly reduced blood glucose and lipid levels than the control groups and showed good therapeutic efficacy in the total clinical effectiveness rate (71.67% vs. 48.33%, *p* < 0.05) and the Toronto Clinical Scoring System (TCSS) scores (5.5 ± 2.4 vs. 6.5 ± 1.7, *p* < 0.05) than the control group. Additionally, FYTL can also increase serum NGF and insulin-like growth factor (IGF) mRNA expression to alleviate neuronal damage.

Another prescription, Fuyuan Huoxue decoction (FYHX), demonstrated significant efficacy in relieving symptoms related to tingling, burning, and numbness in patients compared to the control group, as well as in improving TCSS scores (Zhang and Zhang 2018). A clinical trial reported that FYHX was shown to accelerate muscle nerve conduction speed and regulate blood glucose levels (Zhang 2018).

Clinical evidence supports the therapeutic effects of some proprietary Chinese medicines, developed based on hospital experiences, on DPN. These include Jiangtang Huoluo pill, Jiangtang Tongmailing capsule, Xiongshao capsule, and Danshen and Gegen granule. Compound Xiongshao capsule (XS) (Cui et al. 2018) has shown a significant therapeutic effect in treating DPN by improving NCV, the incubation period, and amplitude of the median and common peroneal nerves, as well as reducing the whole blood high-shear viscosity in patients. Its efficacy is comparable to that of the combination of epoprostenol with beziridine sodium and is superior to that of the combination of furadithiamine with methylcobalamin and epoprostenol monotherapy.

In a clinical study involving the Jiangtang Huoluo pill combined with TCM fumigation (Wu et al. 2019), the total effective rate of the treatment group was 90.0%, significantly higher than that of the control group (*p* < 0.05). Furthermore, both the Jiangtang Tongmailing capsule (Mu 2016) and the Danshen and Gegen granule (Wen 2017) have been shown to aid recovery in DPN patients and are recommended for clinical use.

### Chinese traditional patent medicine

Chinese patent medicines are a category of drugs that have a fixed prescription composition and ratio and are available on the market. Among them, the Mudan granule is currently used to treat DPN. Others currently undergoing clinical studies for DPN include the Mongolian medicine Zhenbao Pill, the compound Dangshen dropping pill, and the Tongxinluo capsule. While these drugs have been available on the market for an extended period, their therapeutic effects on DPN have not been fully validated. Therefore, more preclinical and clinical research is necessary to substantiate its efficacy in treating DPN.

The Mudan granule, composed of nine herbs, was approved in 2008 by the National Medical Products Administration as a specific treatment for DPN. Pharmacological experiments confirmed that Mudan granule can alleviate symptoms of DPN in rats by improving myelinated large fibers, addressing early small nerve fiber lesions, and accelerating NCV (Yang W et al. 2015). Additionally, it has been shown to increase potassium channel kv7.3 mRNA expression and decrease N-type calcium channel protein mRNA expression, aiding in the treatment of painful diabetic peripheral neuropathy (PDPN) (Qi and Yu 2015a).

In the clinical realm, several studies have been conducted to evaluate the efficacy of Mudan granule in DPN patients. In a clinical trial (Qi and Yu 2015b), the treatment group (*n* = 32) received Mudan granule for four weeks, in addition to the standard treatment received by the control group. The results indicated a higher clinical efficacy rate in the Mudan granule group (84.4% vs. 62.5%, *p* < 0.05). It was also found to improve lipid metabolism and antioxidant stress. Another clinical trial observed the effects of Mudan granule administration over eight weeks. Statistical analyses of the left and right common peroneal nerves, as well as the conduction rates of the left and right tibial nerves, showed more significant improvements in the observation group compared to the control group (*p* < 0.05) (Yao 2018; Zhang 2019).

The Mongolian medicine Zhenbao pill (MMZ), which contains 17 herbs, has traditionally been used to treat cerebral hemorrhage, nerve injury, and radiculitis. Recently, it has been discovered that MMZ can also promote the division and proliferation of Schwann cells. Consequently, two research groups (Yi et al. 2019; Zhao 2021) conducted randomized controlled clinical studies to observe the therapeutic effect of MMZ on DPN patients. Although no significant differences were found in blood glucose, lipid levels, TCM syndrome, or Michigan Diabetic Neuropathy Score (MDNS) scores between the MMZ-administered group and the control group, there was a significant increase in MNCV and SNCV of the median and common peroneal nerves in the MMZ group (*p* < 0.05).

In addition, the compound Danshen dropping pill (Yu 2022) and the Tongxinluo capsule (Du 2018), previously used for coronary artery disease and angina pectoris, have been clinically combined with methylcobalamin in the treatment of DPN. This combination was found to alleviate clinical symptoms such as pain and numbness in DPN patients.

### Mechanisms of chinese medicine in treating DPN

**Figure 1.**
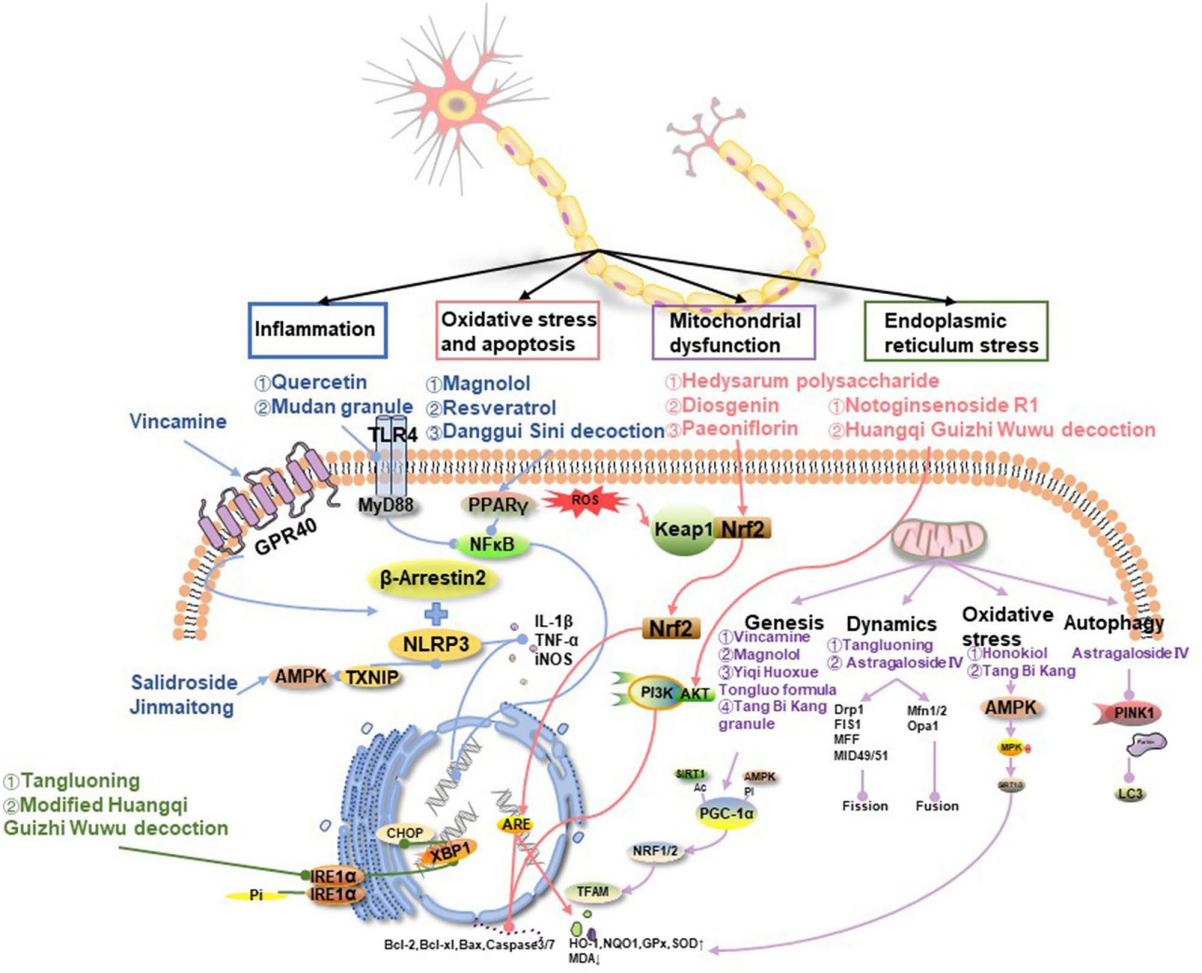
Mechanisms and representative drugs of TCM in treating DPN. Inflammation (blue): alleviated through AMPK, GPR40, TLR4 and PPARγ signal pathways. Oxidative stress and apoptosis (red): inhibit oxidative stress, and apoptosis through Keap1/Nrf2 and PI3K/AKT pathways. Mitochondrial dysfunction (purple): the regulation of mitochondria can be divided into mitochondrial biogenesis, dynamics, oxidative stress and autophagy. Endoplasmic reticulum stress (green): TCM inhibits endoplasmic reticulum stress through the IRE1α/XBP1/CHOP signaling pathway. AMPK: the adenosine 5′-monophosphate (AMP)-activated protein kinase; GPR40: G protein-coupled receptor 40; TLR4: Toll-like receptor 4; PPAR: the peroxisome proliferator-activated receptor; Nrf2: the nuclear factor erythroid 2-related factor 2; PI3K: Class I phosphoinositide 3-kinase; AKT: serine/threonine kinase; Keap1: Kelch-like ECH-associating protein 1; IRE1α: inositol requiring enzyme 1; XBP1: X-box binding protein1; CHOP: CCAAT/enhancer binding protein homologous protein.

There are many mechanisms for treating DPN with traditional Chinese medicine, and the main ones are anti-inflammatory, antioxidation, improvement of mitochondrial function and endoplasmic reticulum stress. These aspects were introduced respectively and summarized in Figure 1.

#### Inflammatory reaction

A growing body of research suggests that neuroinflammation plays a critical role in the pathogenesis of DPN. The systemic inflammatory response, often accompanying insulin signaling dysfunction in hyperglycemic states, activates transcription factors such as nucleotide-binding oligomerization domain-like receptor protein 3 (NLRP3) and NF-κB. This activation triggers a cascade of cytokines and chemokines, leading to an increase in pro-inflammatory cytokines. These cytokines not only contribute to decreased NCV, impaired blood supply to nerves, and degeneration of the axon terminals but also play a significant role in the onset and progression of DPN (Feldman et al. 2019). The mechanism of TCM in treating DPN may involve inhibiting the inflammatory response in nerves by regulating the NLRP3 and NF-κB pathways.

NLRP3 is a central regulator of inflammation and is highly expressed in glial cells and neurons. TCM may exert anti-inflammatory effects by modulating NLRP3. Vincamine, an activator of the G protein-coupled receptor 40 (GPR40), and β-arrestin2, a key regulator of GPR40 signaling, have been implicated in NLRP3 activation. Experimental evidence has shown that in mice treated with vincamine, GPR40 and β-arrestin2 were increased in sciatic nerves and RSC96 cells compared to those of a DPN mouse model. Immunoprecipitation experiments have indicated that β-arrestin2 can bind to NLRP3. Thus, vincamine promotes the binding of NLRP3 to β-arrestin2 and inhibits the expression of pro-inflammatory factors such as IL-1β and TNF-α, as well as the pro-inflammatory enzyme iNOS (Xu JW et al. 2023).

The thioredoxin-interacting protein (TXNIP) is recognized as a central molecule in the inflammatory process leading to the death of insulin-producing cells in the human pancreas. It is upstream of NLRP3, and the TXNIP-NLRP3 complex is essential for the activation of inflammatory vesicles (Sun et al. 2019). Salidroside has been shown to enhance the activity of adenosine 5′-monophosphate (AMP)-activated protein kinase (AMPK), reduce TXNIP levels and inhibit NLRP3 inflammatory vesicle activation. This results in a decrease in serum expression of caspase-1 and IL-1β, thereby alleviating diabetic neuropathic pain (Zheng et al. 2021). Furthermore, Jinmaitong compound has also been found to ameliorate DPN by inhibiting the TXNIP/NLRP3 pathway (Sun et al. 2019).

NF-κB, a transcription factor that mediates immune and inflammatory responses, is composed of subunits including p65, p50, and IκBα. It acts by activating the IκB kinases complex, subsequently releasing heterodimers p65 and p50, which then rapidly enter the nucleus (Yu et al. 2020). High levels of glucose and free fatty acids (FFA) can activate NF-κB and downstream inflammatory pathways, leading to upregulation of pro-inflammatory genes such as IL-1β and TNF-α. Toll-like receptor 4 (TLR4), a transmembrane recognition receptor, recruits myeloid differential protein-88 (MyD88) to activate NF-κB. In studies exploring the mechanism of action of quercetin and Mudan granule in treating DPN, Western Blot analysis (WB) and immunohistochemical experiments showed significant reductions in the expression of TLR4, MyD88, p65, and NF-κB in the sciatic nerve, as well as decreases in inflammatory factors like TNF-α, IL-1β, and IL-6 in the serum and sciatic nerve. These findings suggest that quercetin and Mudan granule may ameliorate typical myelin, axonal, and neuronal damage in rats by inhibiting the inflammatory response through modulatory effects on critical proteins in the TLR-related pathway, including TLR4, MyD88, and NF-κB (Zhao et al. 2021; Zhou and Yao 2021).

The peroxisome proliferator-activated receptor (PPAR) has three isoforms, among which PPARγ is specifically involved in neural tissues, playing a role in glucose and lipid metabolism, insulin sensitivity, and cell growth. Magnolol has been identified as an agonist of PPARγ. Using a PPARγ-specific knockdown mouse model of DPN, it has been shown that PPARγ acts as an upstream pathway of NF-κB, inhibiting NF-κB expression and nuclear translocation, and consequently downregulating TNF-α or iNOS. This leads to attenuation of neuronal damage from the inflammatory response in DPN (Yang J et al. 2022). Similar mechanistic studies on resveratrol and Danggui Sini decoction (Kumar and Sharma 2010; Cheng 2020) for DPN treatment have found that NF-κB protein and mRNA expression, along with inflammatory cascade responses, are downregulated, thus ameliorating elevated levels of TNF-α, IL-6, and COX-2.

#### Oxidative stress and apoptosis

Hyperglycemia-induced oxidative stress is a key contributor to neuronal apoptosis and the subsequent development of neuropathies such as DPN. Studies have established a positive correlation between the occurrence of DPN and the level of oxidative stress. TCM has been shown to potentially inhibit the production of downstream factors involved in oxidative stress. This inhibition is achieved by activating key pathways, namely the nuclear factor erythroid 2-related factor 2 (Nrf2) and the class I phosphoinositide 3-kinase (PI3K)/serine/threonine kinase (AKT) pathways. By modulating these pathways, TCM could alleviate neurotrophic reduction, axonal degeneration, and neuronal apoptosis caused by oxidative stress, thus offering a potential therapeutic approach for DPN.

Nrf2, a crucial transcription factor that regulates cellular oxidative stress, plays a beneficial role in ameliorating oxidative stress and promoting cell survival by maintaining cellular redox homeostasis (Leng et al. 2020). Under normal conditions, Nrf2 is bound to Kelch-like ECH-Associating protein 1 (Keap1) in the cytoplasm. During cellular stress, ROS oxidizes Keap1, leading to the release and nuclear translocation of Nrf2 (He et al. 2022). Nrf2 regulates downstream antioxidant response elements (AREs), including nucleotide adenosine diphosphate hydrolase (NADH), haem oxygenase-1 (HO-1), quinone oxidoreductase-1 (NQO1), and glutamylcysteine ligase (GCLC) (Leng et al. 2020). Additionally, it upregulates anti-apoptotic proteins such as B cell lymphoma-2 (Bcl-2) and Bcl-xL, and downregulates pro-apoptotic protein Bax, thus reducing caspase 3/7 activity and minimizing apoptotic cell death.

Immunofluorescence and WB assays have shown that *Hedysarum* polysaccharide significantly increases Nrf2 protein expression by inhibiting Keap1 expression. This modulation attenuates MDA production, a marker of oxidative stress, in the serum of ob/ob mice, and increases the expression of the glutamate-cysteine ligase catalytic subunit (GCLC), a rate-limiting enzyme in glutathione (GSH) synthesis (He et al. 2022). Moreover, glutathione reductase, an enzyme critical to converting oxidized glutathione disulfide to its reduced form, saw increased mRNA and protein expression under *Hedysarum* polysaccharide treatment, thus mitigating oxidative stress damage to nerves.

Further research into the mechanism of diosgenin in DPN treatment revealed its ability to increase the activity of SOD and glutathione peroxidase (GPx). This is achieved by reversing the decrease in Nrf2 levels under high glucose conditions, thus increasing the expression of downstream HO-1 and NQO1 and reducing MDA levels (Leng et al. 2020). Additionally, paeoniflorin administration to RSC96 cells promoted the upregulation of Nrf2 protein levels, enhancing nuclear translocation and activation of the Nrf2/ARE pathway to alleviate oxidative stress. The Keap1/Nrf2 pathway’s influence on the anti-apoptotic factor Bcl2 was also observed, with increased levels of Bcl2 and decreased levels of Bax and caspase-3 (Yang et al. 2016).

The PI3K/AKT pathway is a classic insulin signaling pathway that plays a crucial role in regulating oxidative stress and apoptosis in neurotoxicity. PI3K is a key regulatory signal in glucolipid metabolism, oxidative stress modulation, and neuroprotection. It activates downstream target proteins, including protein kinase B (AKT), through the phosphorylation of threonine 308 or serine 473 (Tan et al. 2022). In a high glucose state, cellular phosphorylation of PI3K and AKT is inhibited, leading to an increase in pro-apoptosis-related protein expression. Experimental studies have shown that notoginsenoside R1 (NGR1) can activate the PI3K/AKT pathway in a high glucose state. This is achieved by downregulating the expression of miR-503, which is sensitive to high glucose. As a result, NGR1 reduces ROS and caspase-3 expression, improving cell viability, and reducing apoptosis (Wang W et al. 2019).

Similarly, protein expression levels of PI3K, AKT, and phosphorylated AKT (p-AKT) were elevated in the treatment group receiving the Huangqi Guizhi Wuwu decoction. However, when this treatment was combined with LY294002, an inhibitor of the PI3K/AKT pathway, there was a significant decrease in the protein expression of PI3K, AKT, and p-AKT compared to the group treated with TCM alone. The oxidative stress-related indices in this combined treatment group were similar to those observed in the model group. This suggests that the Huangqi Guizhi Wuwu decoction might decrease the ROS content and increase total superoxide dismutase (T-SOD) activity by activating the PI3K/AKT pathway. Therefore, it could effectively enhance the antioxidant capacity of cells and attenuate oxidative stress-induced cellular damage (Gao 2022).

#### Mitochondrial dysfunction

**Figure 2.**
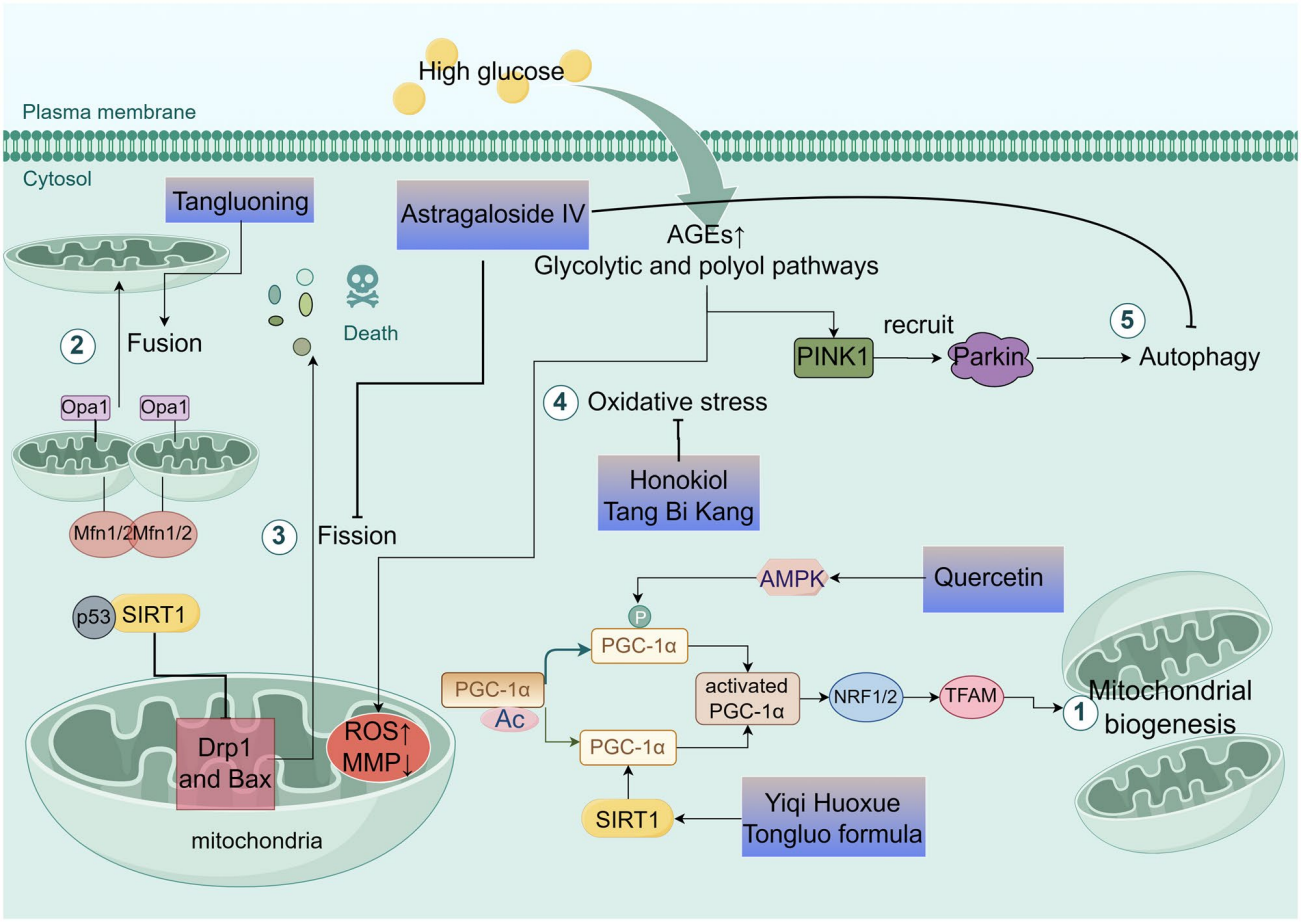
Mechanisms of traditional chinese medicine in treating DPN by improving mitochondrial dysfunction, including mitochondrial biogenesis (①), mitochondrial dynamics (② and ③), mitochondrial oxidative stress (④) and mitochondrial autophagy (⑤) (by figdraw). PGC-1α: PPARγ coactivator-1α; AMPK: the adenosine 5′-monophosphate (AMP)-activated protein kinase; NRF1 and NRF2: nuclear respiratory factors 1 and 2; TFAM: the mitochondrial transcription factor A; SIRT1: Sirtuin 1; Drp1: dynamin-related protein 1; Mfn1/2: mitochondrial protein 1/2; Opa1: Optic atrophy 1; AGEs: advanced glycosylation end products; PINK1: PTEN-inducible kinase 1; parkin: Parkinson’s disease protein.

As the survival of neurons in the peripheral nervous system and their associated distal axons is highly dependent on energy, the core pathogenesis of DPN can be viewed as a state of impaired metabolism and bioenergetic exhaustion (Eid et al. 2023). In high glucose states, mitochondrial dysfunction leads to maladaptive glucose and lipid energy metabolism in human sensory neurons, axons, and Schwann cells (Chandrasekaran et al. 2019). Mitochondria are pivotal in cellular energy metabolism and possess multiple regulatory mechanisms, including mitochondrial biogenesis, dynamics, oxidative stress, and autophagy (Huang et al. 2024). Therefore, Chinese medicine, by targeting these mitochondrial aspects, may play a role in the treatment of DPN and be shown in Figure 2.

Mitochondrial biogenesis, the process of generating new mitochondria from pre-existing ones through division, is regulated by PPARγ coactivator-1α (PGC-1α). This regulation occurs upon activation of PGC-1α through phosphorylation or deacetylation. Activated PGC-1α subsequently stimulates nuclear respiratory factors 1 and 2 (NRF1 and NRF2), which then activate mitochondrial transcription factor A (TFAM), promoting mitochondrial DNA and protein synthesis (Li et al. 2017). Interestingly, PGC-1α activation can be triggered by phosphorylation through AMPK, as well as deacetylation through Sirtuin 1 (SIRT1) (Cardanho-Ramos and Morais 2021). Quercetin, identified as a potential agent for treating DPN, is believed to correct pathological changes such as oxidative stress, reduced ATP production, and mitochondrial morphology damage in DPN rats. It does this by activating the AMPK/PGC-1α pathway and upregulating the expression of AMPK, PGC-1α, NRF1, and TFAM, thus playing a crucial role in protecting neuro mitochondrial biological functions. Furthermore, the compound Yiqi Huoxue Tongluo formula (Zhang L et al. 2022) has been shown to ameliorate DPN by regulating energy metabolism in nerves, as evidenced by the upregulation of Na^+^/K^+^-ATPase activity and increased SIRT1, PGC1α, TFAM mRNA, and protein expression. Additionally, herbal medicines such as magnolol (Yang J et al. 2022), vincamine (Xu JW et al. 2023), and Tang Bi Kang granule (Yu et al. 2023) have demonstrated similar effects.

Mitochondrial dynamics, which include mitochondrial fusion, fission, and degradation, play a crucial role in energy production. Regulation of mitochondrial fission involves primarily dynamin-related protein 1 (Drp1), fission protein 1 (FIS1), mitochondrial fission factor (MFF), and mitochondrial dynamics proteins 49/51 (MID49/51). In contrast, fusion is regulated by mitochondrial proteins 1 and 2 (Mfn1 and Mfn2) for the outer mitochondrial membrane (OMM), and by optic atrophy 1 (Opa1) for the inner mitochondrial membrane (IMM) (Chan 2020). AS-IV (Ben et al. 2021) exhibits a protective effect against mitochondrial damage by regulating the SIRT1/p53 pathway, downregulating the expression of Drp1 and Bax, and upregulating the anti-apoptotic protein Bcl-2. This modulation inhibits mitochondrial fission and concurrently reduces apoptosis. Tangluoning (Zhu et al. 2021) significantly increases ATP and matrix metalloproteinases (MMP) levels in DPN rats by reducing calcium ions. This effect is achieved by upregulation of Mfn1, Mfn2, and Opa1 expression and reduction of the mitochondrial fission-associated Drp1, which improves mitochondrial dyskinesia, enhances the expression of sciatic nerve myelin basic protein (MBP) and myelin protein zero (MPZ), and mitigates neural sheath lesions and demyelination. Moreover, the Mudan granule downregulates the expression of mitochondrial Drp-1, leading to decreased Bax and MDA content, and increased activity of Bcl-2, GSH, and catalase (CAT) indicators (Chen et al. 2017).

Mitochondria are the primary source of ROS in most mammalian cells. In the diabetic state, autoxidative glycosylation, the formation of advanced glycosylation end products (AGEs), and increased activity of the glycolytic and polyol pathways lead to excess ROS production. This excess in mitochondrial ROS contributes to reduced MMP, the release of apoptotic proteins, and, ultimately, mitochondrial dysfunction (Bhatti et al. 2017). When mitochondrial damage and ROS overproduction occur, there is a loss of Schwann cells, myelinated axons, and sensory neurons. This is accompanied by insufficient ATP production, further exacerbating axonal injury and leading to the development of chronic degenerative diseases (Chowdhury et al. 2013). The AMPK/SIRT1 pathway is crucial in countering HG-induced oxidative stress in the nervous system. In RSC96 cells treated with honokiol, there was a reversal of the inhibitory effect of high glucose on AMPK, increased AMPK phosphorylation, and a significant increase in SIRT1 and SIRT3 mRNA and protein levels. This was accompanied by similar enhancements in the protein expression of downstream target genes (SOD1 and SOD2) (Hu M et al. 2023). Similarly, the Chinese medicine Tang Bi Kang has been shown to improve mitochondrial oxidative stress by activating the AMPK/SIRT3 pathway, promoting SOD expression, reducing MDA levels, and increasing the NAD^+^/NADH ratio, as well as ATP synthesis (Yu et al. 2023).

Neural tissues, which are rich in mitochondria, undergo significant changes under oxidative stress under high glucose conditions. Selective autophagy in mitochondria reduces ROS production in these organelles, thus protecting cells from damage. However, neurons are particularly sensitive to autophagy. A sudden increase in autophagic activity can lead to dramatic intracellular changes, enhancing neuronal autophagy and potentially resulting in cell necrosis and neuronal disintegration (Li and Zhang 2023). The PTEN-inducible kinase 1 (PINK1)/Parkinson’s disease protein (Parkin) pathways are among the most extensively studied in mitochondrial autophagy. PINK1 kinase activity is essential for the rapid and efficient recruitment of Parkin to damaged mitochondria (Geisler et al. 2010). Experimental evidence has shown that AS-IV (Wei et al. 2022) significantly downregulates LC3, PINK, and Parkin expression. This downregulation ameliorates oxidative stress and inhibits overactivation of autophagy in Schwann cells.

#### Endoplasmic reticulum stress

The endoplasmic reticulum plays a crucial role in various metabolic processes *in vivo*, including protein synthesis, folding, modification, and transport. When protein misfolding exceeds a certain threshold, it disrupts endoplasmic reticulum homeostasis, inducing endoplasmic reticulum stress (ERS) and leading to neurological injury (O’Brien et al. 2014). During ERS, the accumulation of unfolded proteins causes GRP78 to dissociate from membrane proteins and bind to these unfolded proteins. Inositol requiring enzyme 1 (IRE1α), a transmembrane protein in the endoplasmic reticulum, forms dimers and undergoes autophosphorylation in its free state. The phosphorylated IRE1α (p-IRE1α) splices the mRNA precursor of transcription factor X-box binding protein 1 (XBP1), removing an intron. This sustained expression of XBP1 activates the ERS apoptotic transcription factor CCAAT/enhancer-binding protein homologous protein (CHOP) in the nucleus (Lin and Popko 2009). CHOP can then directly or indirectly induce apoptosis.

Tangluoning and the modified Huangqi Guizhi Wuwu decoction have been found to reduce ERS and improve the structure and function of the sciatic nerve through this pathway. Researchers (Li X et al. 2019) observed that after 12 weeks of administering Tangluoning to DPN rats by gavage, the expression of GRP78 in the treated group was significantly higher compared to the model group, as shown by the WB analysis (*p* < 0.05). Additionally, the expressions of IRE1α/P-IRE1α and XBP-1 were significantly lower in the treated group compared to the model group (*p* < 0.05, *p* < 0.01). These changes in protein content may be related to the observed improvements in sciatic nerve function and myelin structure in rats. Similarly, treatment of DPN with modified Huangqi Guizhi Wuwu decoction (Zhang et al. 2023b) significantly reduced the expression of ERS-related proteins, such as p-IRE1α and CHOP (*p* < 0.05, *p* < 0.01), compared to the model group, according to WB analysis findings. Real-time fluorescence quantitative polymerase chain reaction techniques confirmed these results at the mRNA level for these two proteins. This suggests that the compound could ameliorate neuromyelin structure damage in diabetic conditions by regulating the expression of ERS-related proteins and mRNAs. The potential upstream targets of the compound warrant further investigation.

## Discussion

DPN is a chronic metabolic disorder characterized by a complex etiology and pathogenesis. The risk of microvascular complications begins to manifest even during the prediabetes stage. Patients with prolonged hyperglycemia often develop lipid metabolism disorders, leading to thickening of the capillary basement membranes, endothelial hyperplasia, and swelling of vascular and endothelial cells. These changes contribute to the deposition of glycoproteins in the vessel walls, narrowing of the lumen, microcirculation disorders, and ultimately ischemia and hypoxia-induced neural tissue damage, characteristic of DPN. Nerve damage associated with DPN is evident in both structural and functional changes, including deficiency of constituent proteins and trophic factors.

Preclinical experiments using TCM to examine the structure of nerves in DPN rats have shown that most TCM treatments can improve peripheral fiber degeneration, myelin sheath swelling, and nerve fiber demyelination, resulting in better-aligned nerve fibers. Although these structural improvements are not visually observable in clinical settings, functional evaluations of sciatic nerve performance, including tests for NCV, TPT, and MWT, have shown that TCM can enhance NCV and improve TPT and MWT sensitivity in DPN. Furthermore, certain herbal medicines have been found to increase resistance to injury and regeneration of peripheral nerves by increasing the expression of neurotrophic factors, such as NGF and BDNF.

This review comprehensively summarizes the potential mechanistic pathways through which TCM treats DPN. TCM has been found to alleviate oxidative stress and inhibit apoptosis through the Keap1/Nrf1/HO-1 and PI3K/AKT pathways. Additionally, it can suppress the production of inflammatory factors in nerves through the NLRP3 inflammasome and NF-κB pathways. TCM also enhances mitochondrial function *via* the AMPK/SIRT1/PGC-1α pathway, mitigates endoplasmic reticulum stress by inhibiting the IRE1α/XBP-1/CHOP pathway, and attenuates autophagy-related nerve damage. By modulating one or more of these pathways and their interactions, TCM demonstrates a multifaceted effect, often showing superior preclinical and clinical efficacy compared to Western medications such as methylcobalamin and α-lipoic acid. Future research is essential to unravel the underlying pathogenesis of DPN and to identify the key targets and mechanisms of TCM treatment.

## Conclusions

DPN is a complex metabolic disorder with multifaceted pathogenesis, beginning at the prediabetes stage. TCM has demonstrated efficacy in the treatment of DPN, with promising outcomes observed in the improvement of nerve structure and function. These effects appear to be mediated by mechanisms such as the reduction of oxidative stress and inflammation, and the enhancement of mitochondrial function. TCM, often used in conjunction with Western medications such as methylcobalamin and α-lipoic acid, has demonstrated superior efficacy and a higher safety profile in clinical trials. The future treatment of DPN lies in understanding its pathogenesis and revealing the concrete mechanisms of TCM, which could lead to more effective and integrated treatment.

## Data Availability

The data are available from the corresponding author upon reasonable request.
